# An optimized method of extracting and quantifying active Neutrophil serine proteases from human whole blood cells

**DOI:** 10.1371/journal.pone.0272575

**Published:** 2022-08-31

**Authors:** Jessica Basso, Jimin Zhang, Daniel Lasala, Sasha J. Rose, Kuan-Ju Chen, David Cipolla

**Affiliations:** Insmed Incorporated, Bridgewater, New Jersey, United States of America; Institute of Virology and Immunology: Institut fur Viruskrankheiten und Immunoprophylaxe, SWITZERLAND

## Abstract

**Purpose:**

Neutrophil serine proteases (NSPs) are implicated in numerous inflammatory diseases. Thus, a robust methodology to monitor and quantify NSPs is important to study disease progression and evaluate the effect of pharmacological interventions. A comparison of the various methods used to extract NSPs from neutrophil granulocytes has not been published, providing the impetus to conduct this method optimization and comparison study.

**Methods:**

Two NSP recovery methodologies were evaluated on samples from five human donors: zymosan stimulation and cell pellet extraction. For the zymosan stimulation method, 1 mL donor blood was added to zymosan and samples were incubated at 37°C for 30 min while shaking. Samples were then centrifuged, and the plasma was collected for quantitation of NSP activity. For the cell pellet extraction procedure, 2 mL whole blood samples were centrifuged into white blood cell pellets following red blood cell lysis. To each pellet, three sequential lysis steps were performed using either 0.05% Nonidet P-40 Substitute (NP40) or 0.02% Triton X-100 lysis buffers under agitation followed by centrifugation. NSP activities were quantified using an exogenous peptide substrate specific to each of the three NSPs being analyzed: neutrophil elastase, cathepsin G, and proteinase 3.

**Results and discussion:**

The zymosan stimulation method resulted in lower recovery of active NSPs and was unable to stimulate significant release of active cathepsin G. In contrast, the NP40 pellet extraction method showed consistent inter-donor NSP release with greater recoveries of active NSPs than the Triton method or the zymosan stimulation method. Overall, the pellet extraction procedure provided 13.3-fold greater recovery of active neutrophil elastase, 283-fold greater recovery of active cathepsin G, and 2.9-fold greater recovery of active proteinase 3 than the zymosan method.

**Conclusion:**

The NP40 cell pellet extraction method resulted in greater extraction of active NSPs compared to the other methods investigated here, which may allow for a more accurate and complete biomarker profile when evaluating human clinical samples.

## Introduction

Neutrophil elastase (NE), proteinase 3 (PR3), and cathepsin G (CatG) are structurally related enzymes that are stored within the neutrophil cytoplasmic azurophilic granules and are collectively known as neutrophil serine proteases (NSPs) [1,2]. NSPs are synthesized as inactive pre-pro-proteins during granulocyte development and are converted to active proteins by dipeptidyl peptidase 1 (DPP1, also known as cathepsin C) during the neutrophil maturation process in bone marrow. NSPs play a foremost role in pathogen destruction, contributing to inflammatory regulation and modulation, and are known to operate via at least three pathways: When neutrophils engulf and phagocytose bacteria, NSPs gain access to the pathogen following fusion of the neutrophil granules with the phagosome. Secondly, upon neutrophil activation, neutrophil granules can also be released into the extracellular milieu via exocytosis. A third mechanism whereby NSPs can be found in the extracellular space is via the formation of neutrophil extracellular traps (NETs), which are extruded from activated neutrophils and comprised of chromatin decorated with histones and proteases [3]. The process of NET formation is complex and incompletely understood, but has been reported to involve the translocation of NE from the azurophilic granules to the cell nucleus where it is thought to cleave histones, thereby facilitating chromatin decondensation, and subsequent extrusion to the extracellular space to immobilize pathogens [4]. Of concern in chronic inflammatory diseases, neutrophil accumulation and activation can result in excess secretion of active NSPs causing damaging inflammation and tissue matrix destruction [1,2]. Given the involvement of NSPs in host defense and their importance in studying and characterizing disease, optimal extraction and accurate measurement of these biomarkers are essential.

To extract NSPs for quantitation, the cells must be either (1) stimulated causing their release, or (2) both the cell and granules must be lysed. Zymosan is known to stimulate and induce inflammatory responses of leukocytes, including triggering the release of NE–an inflammatory protease [5,6]. This method of stimulation has also been used in Phase I and Phase II clinical trials where NE activity was measured in the blood following zymosan stimulation [5,7]. This stimulation method requires accurate preparation of the zymosan and immediate stimulation and supernatant collection, and the length of stimulation can also affect enzyme recovery. Clinical technicians often do not have the scientific training for the precision required by this protocol, which may impact their ability to implement this procedure in large-scale clinical trials. While zymosan-stimulation is a recognized clinical-standard methodology, some experiments have shown opsonized zymosan to be a poor stimulator of elastase from human neutrophils compared to other inducers such as N-formyl-L-methionyl-L-leucyl-L-phenylalanine (fMLP) [8]. Thus, it is important to understand how effective the zymosan-stimulation methodology is for recovery of NSPs and whether there are improved methodologies for quantitation of NSPs from blood samples in clinical trials.

Traditional methods of cell lysis include physical disruption and chemical methods [9]. Lysis methods may combine both a chemical surfactant and physical agitation to break open the cell membrane and expose the NSPs from white blood cells (WBCs) collected from whole blood. Importantly, the choice of surfactant may affect NSP recovery and/or interfere with the method of measurement [9]. Generally, surfactants can be classified into three categories: ionic, non-ionic, and zwitterionic. Ionic surfactants are known to denature proteins by forming electrostatic and hydrophobic interactions that disrupt the protein’s tertiary structure [10]. Non-ionic surfactants, such as Triton X-100 and Nonidet P-40 (NP40) are non-denaturing, breaking only the lipid-lipid and lipid-protein interactions while maintaining protein-protein interactions. Lastly, zwitterionic surfactants, such as CHAPS, combine the properties and effects of ionic and non-ionic surfactants. They have no net charge but can denature proteins present in the sample [9]. Thus, non-ionic surfactants are ideal to disrupt the neutrophil cell membrane and the lipid bilayer of intracellular granules allowing release of NSP proteins without denaturing them.

Two non-ionic surfactants, Triton X-100 and NP40 usually buffered in Tris at neutral pH, have been used to recover NSPs from a range of cells including circulating neutrophils [10–17]. A range of concentrations of Triton X-100 and NP40 have been reported in NSP lysing buffers. Triton X-100 at 10% has been used to lyse primary bone-marrow-derived CD34^+^ neutrophil progenitor cells [11], or circulating neutrophils isolated from Sprague-Dawley rat blood and leukocytes from rat bone marrow [12]. Triton X-100 at 0.2% was used to lyse the pro-monocytic cell line U937 and bone marrow extracts from male BALB/c mice [13] or from circulating neutrophils in healthy male human subjects in a Phase 1 clinical trial [16]. NP40 at 1.0% was used to lyse U973 cells [14], at 0.5% was used to lyse TALL-104 cells [14], at 0.2% was used to lyse U937 and EcoM-G cells [15] and circulating leukocytes from *in vivo* rat studies [14], and at 0.05% was used to lyse differentiated hematopoietic stem cells isolated from umbilical cord blood as well as circulating neutrophils from patients with Papillon-Lefèvre Syndrome [17].

To our knowledge, no head-to-head evaluation of these methodologies to extract NSPs from neutrophil granulocytes has been published and it is unclear whether a specific surfactant concentration is ideal. Therefore, an investigation into the methodologies of extraction was undertaken to determine the best extraction method for researchers and clinical laboratories to implement moving forward. Triton X-100 (at 0.02%, 0.2%, 1%, and 10%) was evaluated in preliminary NSP extraction studies with the outcome that 0.02% Triton X-100 provided superior extraction with minimum assay interference (S1 Fig). Regarding NP40, since gelling of lysate was observed in our lysis assay protocol using higher NP40 concentrations, 0.05% NP40 was chosen to minimize gelling and improve NSP recovery. The effectiveness of these two lysis buffers for NSP extraction from blood samples of five human donors was compared to that obtained with zymosan stimulation. Additionally, for the 0.05% NP40 lysis buffer, which was superior to the 0.02% Triton X-100 lysis buffer in our preliminary evaluation, various modifications to the processing conditions were also evaluated to determine the method robustness to changes and whether simplifications to the clinical protocol could be introduced.

## Materials and methods

The methods section summarizes the sample processing procedures performed on fresh whole blood prior to NSP extraction and details the zymosan stimulation method that was utilized in human clinical trials [5,7], and which was followed in this study. It describes the various cell pellet extraction procedures utilizing the 0.02% Triton X-100 buffer or the 0.05% NP40 NSP buffer [17]. The enzymatic kinetic assays developed for NSP measurement, along with the data and statistical analysis of NSP quantitation, are also described. A summary of the lysis solutions and reagent information are detailed in Table 1.

**Table 1:** Lysis solutions and assay reagent information.

	Reference Name	Formulation
Lysis Buffers	0.02% Triton X-100	1xPBS + 150 nM NaCl + 0.02% Triton X-100 (CAS No. 9036-19-5)
	NP40	50 mM HEPES buffer + 0.75M NaCl + 0.05% Nonidet P-40 Substitute (Sigma, Cat. No. 74385)
Assay Reagents & Buffers	Tris Assay Buffer	100 mM Tris (Sigma, Cat. No. T7818), 100 mM NaCl in H_2_O, pH 7.5
	Enzyme Buffer	0.05% Triton X-100 (CAS No. 9036-19-5) in Tris Assay Buffer
	NE Substrate Diluent	2M NaCl in Tris Assay Buffer
	NE Substrate	Methoxysuccinyl-ala-ala-pro-val-AMC (Sigma, CAS No. 72252-90-5)
	PR3 Substrate	Abz-VADCADQ-Lys(DNP) (Genescript custom synthesis)
	CatG Substrate	Suc-AAPF-pNA (Sigma, Cat. No. S7388)
	Human NE Stock Protein	Elastase from Human Leukocytes (Sigma, Cat. No. E8140, 100 ng/μL in 50% glycerol-PBS v/v)
	Human PR3 Stock Protein	PR3 from Human Neutrophils (Sigma, Cat. No. SRP6309, 100 ng/μL or 50 ng/μL in 50% glycerol-PBS v/v)
	Human CatG Stock Protein	CatG from Human Leukocytes (Sigma, Cat. No. C4428, 100 ng/μL in 50% glycerol-PBS v/v)

### Ethics statement

The study was approved by Insmed’s Ethics Committee under protocol RDL-20-001. The study was fully and carefully explained to the five donor participants, and they clearly understood the nature, risks, and benefits of their participation in this experimental clinical research study. No minors were included in the study. Written consent was informed prior to participation in the study.

### Blood collection procedure

Volunteers were selected from those who were at low risk of spreading any known virus. Blood was collected from five (5) human donors at the same location of the laboratory where the samples were processed immediately after collection. A total of 24 mL whole blood was obtained from each human volunteer into the citrated blood tubes and inverted 4 times. Blood samples from each donor were homogenously separated into aliquots of 1 mL for the zymosan method and aliquots of 2 mL for the cell pellet extraction method.

### Zymosan stimulation

Two zymosan preparation conditions were evaluated in this study: either boiled as per the manufacturer’s instructions or un-boiled. The manufacturer’s zymosan preparation protocol was modified from 30 minutes of boiling to 15 minutes.

The zymosan suspension (10 mg/mL) was prepared by dissolving 50 mg zymosan (Sigma, Cat No. 4250) in 5 mL 0.9% saline on the day of the study. A 1 mL portion of this zymosan suspension was transferred to a microcentrifuge tube and boiled in a water bath for 15 minutes, centrifuged (20,000 x *g* for 1 minute) to pellet the zymosan, and the supernatant was discarded. The pelleted zymosan was resuspended in 1 mL 0.9% saline and vortexed to mix. Next, 100 μL of the zymosan suspension (un-boiled and boiled) was aliquoted into microcentrifuge tubes requiring stimulation (2 tubes/donor) and 100 μL of 0.9% saline was aliquoted to the negative control tubes (3 tubes/donor).

Immediately after donor blood collection, 1 mL fresh whole blood per donor was transferred into the appropriate microcentrifuge tube prefilled with either zymosan (boiled or un-boiled) or saline (as negative background control) and inverted four (4) times to mix. Samples were then incubated for 30 minutes in a shaking incubator at 37°C at 100 rpm. After incubating, samples were centrifuged (2,000 x *g* for 10 minutes at 4°C) and 100 μL plasma aliquots were collected into fresh tubes and stored on ice until being transferred to a -80°C freezer prior to NSP assay. There was a total of seven (7) tubes per donor: two (2) tubes containing un-boiled zymosan, two (2) tubes boiled zymosan, and three (3) tubes saline. The third triplicate of saline was later pooled with the other donors and was used to create a standard diluent to match the sample matrix. This processing procedure takes approximately 45 to 60 minutes using a fully prepared zymosan solution.

### Cell pellet extraction sample processing

#### Red blood cell lysis of donor samples

Prior to performing NSP extractions, donor whole blood samples were processed into WBC pellets by lysing 2 mL whole blood with 40 mL 1x RBC Lysis Buffer (Abcam, Cat No. Ab204733), inverting 5 times, and incubating at room temperature for 20 minutes. Since each donor has a distinct NSP recovery profile, WBC pellets were generated for each donor so that different processing conditions could be accurately compared. The lysed whole blood samples in the 50 mL centrifuge tubes were then centrifuged (400 x *g* for 5 minutes at 4°C), followed by careful decantation of the liquid (supernatant) without disrupting the WBC pellet. The supernatants were not retained. Two 50 mL centrifuge tubes per donor containing the retained white blood cell pellets were frozen and stored at -80°C prior to subsequent NSP extraction using 0.05% NP40 lysis buffer (Condition A). Processing condition A represents the methodology that would be the simplest to follow at the clinical site by removing the pellet washing step.

The remaining pellets were washed with 40 mL 0.9% saline, inverted 5 times to mix, centrifuged again (400 x *g* for 5 minutes at 4°C), followed by careful decantation of the liquid (supernatant) without disrupting the WBC pellet. The supernatants were not retained. The centrifuge tubes containing the retained WBC pellet were frozen and stored at -80°C prior to subsequent NSP extraction (Conditions B, C, and D). Processing conditions B and C also utilized 0.05% NP40 lysis buffer while processing condition D utilized 0.02% Triton X-100 lysis buffer. Processing conditions C and D sampled all three lysis cycles prior to pooling to allow for an analysis of the relative NSP recovery in each of the three lysis steps. This may provide a determination of whether further extraction steps are warranted to meaningfully increase extraction recovery. Refer to Table 2 for a summary of the cell pellet processing and lysis conditions. This processing procedure takes approximately 30 to 45 minutes.

**Table 2:** Summary of cell pellet processing and lysis for each donor.

		Unwashed Pellets	Washed Pellets		
	Process Condition:	A (n = 2)	B (n = 1)	C (n = 1)	D (n = 1)
**RBC Lysis**	**RBC Lysis**	Yes			
	**Post RBC Lysis Wash**	No	Yes with 0.9% Saline		
**WBC Pellet Lysis**	**Wash with TAB** *	Yes	No		
	**Lysis Buffer**	0.05% NP40			0.02% Triton
	**Analysis of each of 3 lysis cycles**	No		Yes	

*TAB = Tris Assay Buffer.

#### White blood cell pellet lysis of donor samples

WBC pellets were processed according to Table 2. Individual samples were thawed at room temperature. Following thawing, all steps of this procedure were performed on ice. Duplicate pellets for condition A that were not previously washed during the RBC lysis, were washed with 1 mL Tris Assay Buffer (100 mM Tris, 100 mM NaCl in H_2_O, pH 7.5), centrifuged (16,000 x *g* for 3 minutes at 4°C) to pellet debris, and the washes were collected in separate tubes. All pellet samples were then lysed via mechanical pipetting of each sample twenty (20) times with 1 mL lysis buffer–either 0.05% NP40 or 0.02% Triton X-100 (Table 1). A multichannel pipette was used to ensure consistent mechanical lysis conditions for each donor. Post lysis, samples were centrifuged (16,000 x *g* for 10 minutes at 4°C) to pellet the cell debris. The lysis supernatants were collected, and this process was repeated for a total of three lysis rounds. For conditions C and D, 250 μL samples were removed from each of the 3 lysates. For all conditions, the lysates were pooled for each pellet sample, and for condition A, the pellet wash was additionally assayed to account for NSPs that may have been released in the wash buffer following freeze/thaw but prior to lysis. Pooled and individual lysis fractions were stored at -80°C for subsequent NSP testing. This procedure takes approximately 3 to 4 hours.

### NSP activity quantitation

The kinetic assays to quantify the activity of each NSP takes approximately 45 minutes to prepare the plate and 1.5 hours to read the plate.

#### Kinetic NE assay

NE activity was measured in an enzymatic assay using an exogenous peptide substrate with high specificity for NE. Cleavage of the substrate by NE present in the sample generated the fluorophore reaction product, 7-amino 4-methyl coumarin (AMC). The initial rate of this reaction is proportional to the amount of active NE in the sample and was measured in relative fluorescence units (RFU) and converted to concentration of active NE.

Standards were created via serial dilution of stock human NE protein (Sigma, Cat. No E8140-1UN) in a standard diluent that matched the ratio of lysis buffer or plasma to enzyme buffer (sample diluent) of the sample dilutions on the plate. Sample dilutions were created on the plate using enzyme buffer as the diluent and/or run neat. DMSO or NE inhibitor (Abcam, Cat. No ab142154, final concentration 80 μM) in Tris Assay Buffer was added to all wells and allowed to incubate at 37°C for 15 minutes. Addition of DMSO was used as a placeholder in case future testing required addition of inhibitors for subtraction of nonspecific activity cleavage. Note that 0.25% v/v antifoam can be added to the inhibitor/DMSO Tris Assay Buffer diluent for testing with cell pellet lysate samples to reduce potential errors due to bubble formation. Following incubation, NE substrate (Methoxysuccinyl-ala-ala-pro-val-AMC, Sigma, CAS No. 72252-90-5, final concentration 100 μM) diluted in NE substrate diluent was added to the sample and control wells in that order, and briefly mixed via pipetting 2–3 times. The plate was immediately read at 350/450 nm (Ex/Em) post substrate addition in kinetic mode every 5 minutes for 1.5 hours at 37°C on the BioTek Synergy Neo or BioTek Synergy H1M plate reader, both paired with Gen5 Software. The raw data was exported into an Excel file for data analysis (see *Data Analysis* methods below).

#### Kinetic PR3 assay

This assay follows the same principles as the *Kinetic NE Assay* (see above), except using exogenous peptide substrate specific to PR3 instead of NE. Cleavage of the substrate by PR3 present in the sample generated the fluorophore reaction product, 2-Aminobenzoyl or Anthraniloyl (Abz). The initial rate of this reaction is proportional to the amount of active PR3 in the sample and was measured in RFU and converted to concentration of active PR3.

Standards were created via serial dilution of stock human PR3 protein (Sigma, Cat. No SRP6309-25UG) in a standard diluent which matched the ratio of lysis buffer or plasma to enzyme buffer (sample diluent) of the sample dilutions on plate. Sample dilutions were created on the plate using enzyme buffer as the diluent and/or run neat. DMSO or PR3 inhibitor (Abcam, Cat. No ab146184, final concentration 500 μM) in Tris Assay Buffer was added to all wells and allowed to incubate at 37°C for 15 minutes. Addition of DMSO was used as a placeholder in case future testing required addition of inhibitors for subtraction of nonspecific activity cleavage. Note that 0.25% v/v antifoam can be added to the inhibitor/DMSO Tris Assay Buffer diluent for testing with cell pellet lysate samples to reduce potential errors due to bubble formation. Following incubation, PR3 substrate (Abz-VADCADQ-Lys(DNP), GenScript synthesized, final concentration 100 μM) diluted in Tris Assay Buffer was added to the sample and control wells in that order, and briefly mixed via pipetting 2–3 times. The plate was immediately read at 340/430 nm (Ex/Em) post substrate addition in kinetic mode every 5 minutes for 1.5 hours at 37°C on the BioTek Synergy Neo or BioTek Synergy H1M plate reader. The raw data was exported into an Excel file for data analysis (see *Data Analysis* methods below).

#### Kinetic CatG assay

This assay follows the same principles as the *Kinetic NE and PR3 Assays* (see above), except using exogenous peptide substrate specific to CatG. Cleavage of the substrate by CatG present in the sample generated the chromophore reaction product, p-Nitroaniline (pNA). The initial rate of this reaction is proportional to the amount of active CatG in the sample and was measured in absorbance (ABS) units and converted to concentration of active CatG.

Standards were created via serial dilution of stock human CatG protein (Sigma, Cat. No C4428-.25UN) in a standard diluent which matched the ratio of lysis buffer or plasma to enzyme buffer (sample diluent) of the sample dilutions on plate. Sample dilutions were created on the plate using enzyme buffer as the diluent and/or run neat. DMSO or CatG inhibitor (Cayman Chemical, Cat. No 14928, final concentration 200 μM) in Tris Assay Buffer was added to all wells and allowed to incubate at 37°C for 15 minutes. Addition of DMSO was used as a placeholder in case future testing required addition of inhibitors for subtraction of nonspecific activity cleavage. Note that 0.25% v/v antifoam can be added to the inhibitor/DMSO Tris Assay Buffer diluent for testing with cell pellet lysate samples to reduce potential errors due to bubble formation. Following incubation, CatG substrate (Suc-AAPF-pNA, Sigma, Cat No. S7388, final concentration 400 μM) diluted in Tris Assay Buffer was added to the sample and control wells in that order, and briefly mixed via pipetting 2–3 times. The plate was immediately read at 405 nm post substrate addition in kinetic mode every 5 minutes for 1.5 hours at 37°C on the BioTek Synergy Neo or BioTek Synergy H1M plate reader. The raw data was exported into an Excel file for data analysis (see *Data Analysis* methods below).

### Data analysis

#### Data analysis method for kinetic NSP assays

In brief, one of two methods were used to analyze the results of the kinetic reads; these methods were compared to ensure consistency of results.

Readings from BioTek’s Synergy Neo and H1 plate reader with Gen5 software were incorporated into an Excel file for data analysis. From the raw data, the linear portion of the kinetic slopes (S2 Fig) was either (1) visually determined and calculated using Excel’s slope formula or (2) automatically determined and calculated using an internally developed macro-Excel program. Standard curves were created using the standard slope values and their respective known concentrations. Multiple standard curves were created if multiple standard diluents were used in the assay to match the various sample dilutions. The unknown sample concentrations were then calculated using the second-degree polynomial line of best fit formula from the appropriate standard curves. Sample concentrations were corrected for dilution, nonspecific activity (defined as remaining activity with inhibitor) was subtracted if present, and duplicates were averaged. Values are reported as mass concentrations of active NSP per mL whole blood. Mass concentration units were reported instead of molar concentration units, which are traditionally used for activity measurements, to be consistent with recent usage for the measurement of active NSP biomarkers in biological samples [18–20]. For brevity, the “concentration of active NSP” is often shortened to “NSP activity.” The molecular weights of NE, PR3, and CatG are comparable at 29,500, 29,000, and 29,000 g/mol, respectively, so comparison of their mass concentrations also provides a relative sense for their molar ratios.

### Statistical analysis

Statistical analyses were performed using GraphPad Prism 8.2.1.

A two-way ANOVA followed by a Tukey’s multiple comparison test was performed comparing the average stimulant (saline, zymosan, boiled zymosan) NSP level for all groups. The alpha value was set at 0.05 for analysis.

A repeated measures one-way ANOVA followed by Sidak’s multiple comparison test was performed comparing the donor’s NSP levels when processed according to unwashed pellet condition A procedure, which results in a dual assay, to an optimized single assay processing procedure with the same lysis buffer (Condition B). Additionally, the post-hoc analysis also compared the NSP levels of pellets that were processed in a similar fashion but lysed with different lysis buffers (NP40 (Condition C) or Triton X-100 (Condition D)), and pooling lysate (Condition B) with separated and summed lysate fractions (Condition C). The alpha value was set at 0.05 for analysis. Refer to Table 2 for cell pellet processing and lysis conditions.

## Results

### Zymosan stimulation method

Zymosan stimulation was previously used to investigate a drug candidate’s effect on NE in the blood [5,7] and their protocol was followed for preparing the zymosan, stimulating the whole blood, and collecting the plasma for NSP testing (see *Methods*). However, their zymosan preparation instructions did not state whether the zymosan was boiled, and if it was not boiled, then their process would have differed from the manufacturer’s instructions. Thus, the un-boiled and boiled zymosan preparations were evaluated. Plasma samples were diluted 1:1 in enzyme buffer for the NE assay. When compared to enzyme buffer alone, the 1:1 plasma-enzyme buffer standard curve showed high assay interference of approximately 95% (S3 Fig). However, NE activity was still able to be quantified using the 1:1 plasma-enzyme buffer matched standard after correcting for interference.

**Fig 1:**
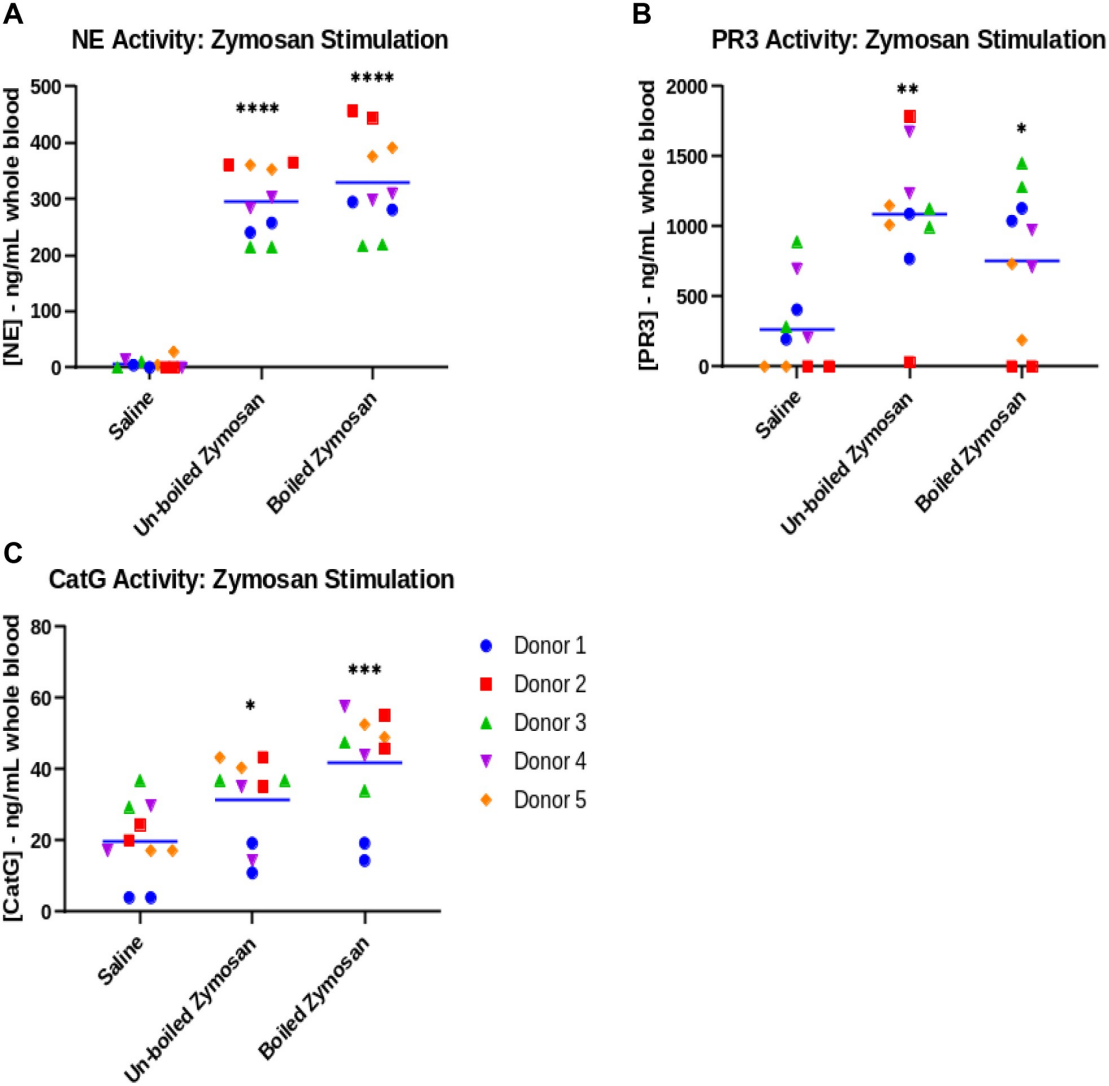
NSP activity recovered after zymosan stimulation of donor whole blood. Data are plotted as the grand mean (blue bar) of experimental repeats (n = 2) plotted for all donors (n = 5). Tukey’s multiple comparison test was used for statistical analysis of groups. *, P < 0.05 vs saline; **, P < 0.01 vs saline, ***, P < 0.001 vs saline, ****, P < 0.0001 vs saline.

NSP recoveries for the zymosan-stimulation method are shown in Fig 1 and S1 Table. Negative control samples (saline stimulated plasma) showed negligible NE activity of 6.13 ± 6.27 ng/mL whole blood versus un-boiled zymosan (294.99 ± 65.04 ng/mL whole blood) and boiled zymosan (328.47 ± 90.07 ng/mL whole blood). Recovery of active NE was affected by whether zymosan was boiled (P = 0.0264) but displayed relatively consistent inter-donor activity for both boiled and un-boiled zymosan preparations (Fig 1A). Donor repeats for stimulated samples showed good agreement with a coefficient of variation (CV) less than 5% for each donor, suggesting that the assay is repeatable for NE (S2 Table).

For the PR3 assay, plasma samples were diluted ten-fold in enzyme buffer to reduce the potential for plasma interference, and their values remained within the range of the standard curve. When compared to enzyme buffer alone, the buffer standard curve still showed high assay interference of approximately 85% (S3 Fig). However, PR3 activity was able to be quantified using the matched standard after correcting for interference. Negative control samples (saline) showed relatively high and variable PR3 background activity (269 ± 266 ng/mL whole blood) versus group mean PR3 values of 1086.96 ± 221.17 ng/mL whole blood (P = 0.0036) and 751.00 ± 535.24 ng/mL whole blood (P = 0.0135) for un-boiled zymosan and boiled zymosan, respectively. Zymosan-stimulated recovery of active PR3 was unaffected by whether zymosan was boiled (P = 0.2961) but displayed significant variability in donor replicates (Fig 1B and S2 Table). Considering that the experiment replicates were performed on the same day with the same batch of reagents and stimulants, this variability suggests that the zymosan method is not optimal for extracting PR3.

Plasma interference was minimal in the CatG activity assay when the plasma samples were diluted 1:1 in the enzyme buffer (S3 Fig). Negative control samples (saline) showed low CatG background activity (19.99 ± 10.68 ng/mL whole blood) but zymosan stimulation only modestly increased CatG activity to 31.54 ± 11.30 ng/mL whole blood (P = 0.0111) and 41.92 ± 14.68 ng/mL whole blood (P = 0.0009), for un-boiled zymosan and boiled zymosan, respectively. Recovery of active CatG was unaffected by whether zymosan was boiled (P = 0.07371) and displayed relatively consistent inter-donor activity (Fig 1C and S2 Table).

### Cell pellet extraction method

For the cell pellet method, previous experiments in our laboratory suggested that residual RBC lysate could interfere with the NP40 lysis buffer, especially for PR3, and therefore should be “washed off”; thus, this step was incorporated into conditions B, C and D. However, insertion of this additional step at a clinical site would lengthen their processing time and could introduce compliance issues. Thus, condition A avoids this step but incorporates a wash step after thawing of the pellet, which could be incorporated at an analysis lab recognizing that this would increase (by approximately double) the processing and NSP quantitation efforts. Conditions B, C and D were identical, except that 0.05% NP40 was used in conditions B and C and 0.02% Triton X-100 was used in condition D. Conditions C and D additionally assayed individual extraction cycles to allow for an estimate of the value of incorporating additional extraction cycles into the process. Table 2 summarizes the cell pellet processing and lysis information.

#### NSP recovery from pooled lysates

**Fig 2:**
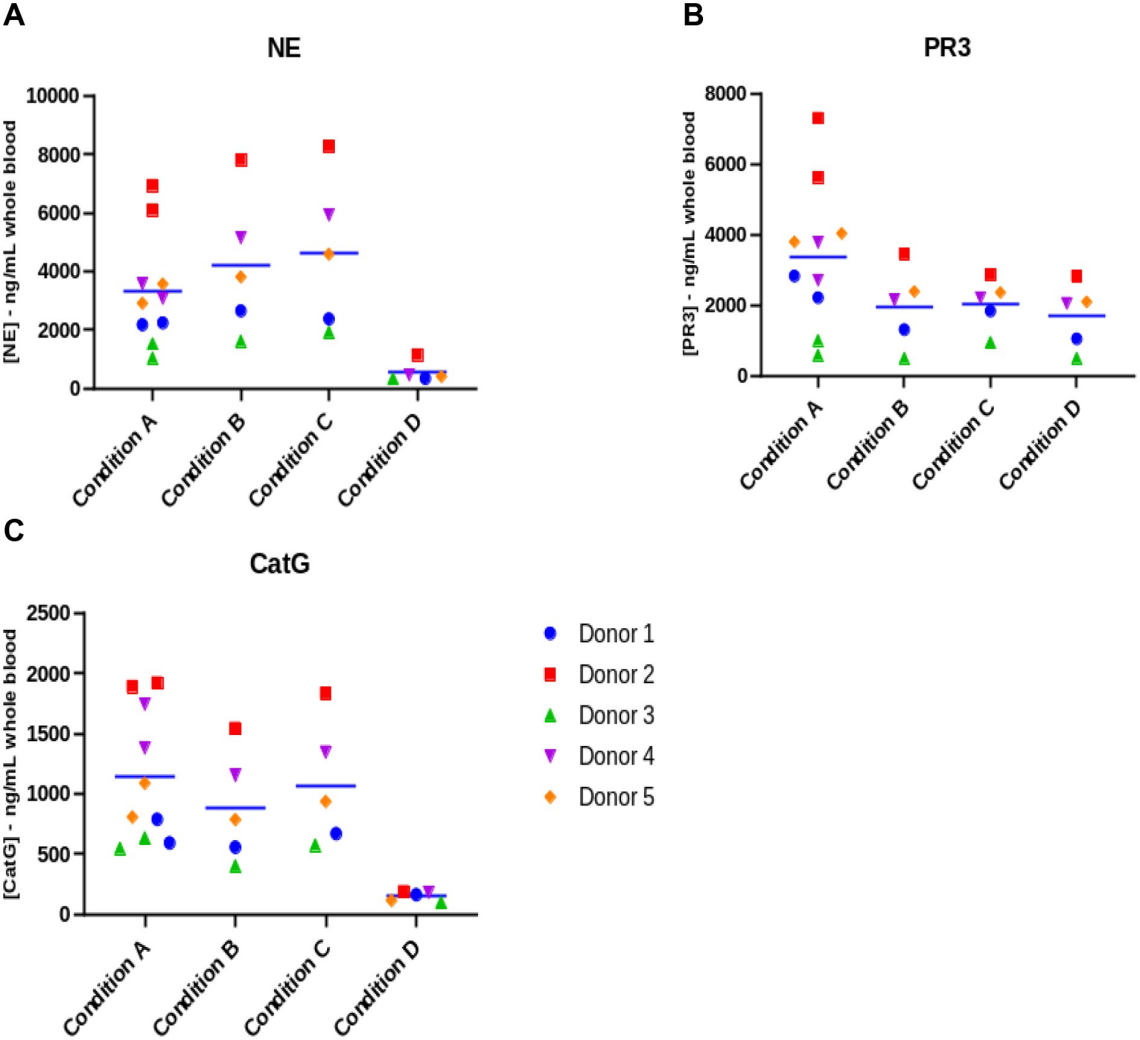
NSP activity recovered for various processing conditions. Data are plotted as the grand mean (blue bar) of experiment repeats (n = 2 for condition A, n = 1 for all other conditions) for all donors (n = 5). For conditions C and D, data is graphed as the summation of individual lysate fractions. Sidak’s multiple comparison test was used for statistical analysis of groups; however, statistical findings are not indicated on the graphs.

The NSP recoveries are shown in Fig 2 and S3 Table for the three NP40 lysis conditions and the single Triton X-100 lysis condition (condition D). All three NP40 lysis conditions produced comparable inter-doner results for NE, PR3, and CatG. Considering that condition A is the result of the assay of two sample fractions (wash and lysate), donor repeats for the condition A replicates showed good agreement with a coefficient of variation (CV) typically less than ~20% for each donor, suggesting that the cell pellet generation procedure and subsequent NSP quantitation are repeatable for all three NSPs (Table 3).

**Table 3:** Donor replicates of NSP activity after cell pellet lysis using Condition A processing (NSP ng/mL whole blood, mean and %CV for each donor replicate, n = 2).

		*Donor 1*		*Donor 2*		*Donor 3*		*Donor 4*		*Donor 5*	
		Mean	%CV	Mean	%CV	Mean	%CV	Mean	%CV	Mean	%CV
***Condition A (n = 2)***	**NE**	2211.5	2.2	6520.4	9.0	1290.0	26.4	3332.2	10.4	3241.9	14.3
	**PR3**	2522.8	17.0	6466.6	18.3	799.7	36.8	3251.9	23.2	3916.9	4.4
	**CatG**	691.0	20.2	1905.9	1.1	591.7	10.2	1563.2	16.4	947.7	21.0

The NE and CatG levels were meaningfully greater using NP40 (conditions A, B and C) for lysis than using Triton X-100 (condition D). NP40 lysis (condition C) showed approximately 8.5-fold greater recovery of active NE compared to Triton X-100 lysis (P = 0.0507) (Fig 2A), approximately 1.2-fold greater recovery of active PR3 compared to Triton X-100 lysis (P = 0.1606) (Fig 2B), and approximately 7.1-fold greater recovery of active CatG compared to Triton X-100 lysis (P = 0.0406) (Fig 2C).

The ratio of total NSP recovery for condition A, which lacked the post RBC lysis washing step but incorporated a pellet washing step post freeze-thaw, was compared to that for condition B, which is simpler for NSP quantitation (Table 4). For condition B, the mean recovery of NE was 27% greater (P = 0.1026), the mean recovery of PR3 was 42% lower (P = 0.0937) and the mean recovery of CatG was 23% lower (P = 0.0327) compared to condition A. While the reduced recovery of both PR3 and CatG is a potential concern using the simplified assay procedure associated with condition B, the relative ratio of PR3 and CatG recoveries across the five donors was maintained suggesting that the assay will be able to determine changes in both PR3 and CatG levels in response to a drug candidate’s treatment effect (Table 4).

**Table 4:** NSP activities recovered from Condition A compared to Condition B.

	Recovery of Active NSPs (Ratio of Condition A to Condition B)		
	NE	PR3	CatG
**Donor 1**	0.83	1.93	1.24
**Donor 2**	0.83	1.87	1.23
**Donor 3**	0.80	1.59	1.47
**Donor 4**	0.65	1.51	1.35
**Donor 5**	0.85	1.64	1.21
**Average ± SD**	**0.79 ± 0.08**	**1.71 ± 0.18**	**1.30 ± 0.11**

#### NSP recovery from each lysis cycle

**Fig 3:**
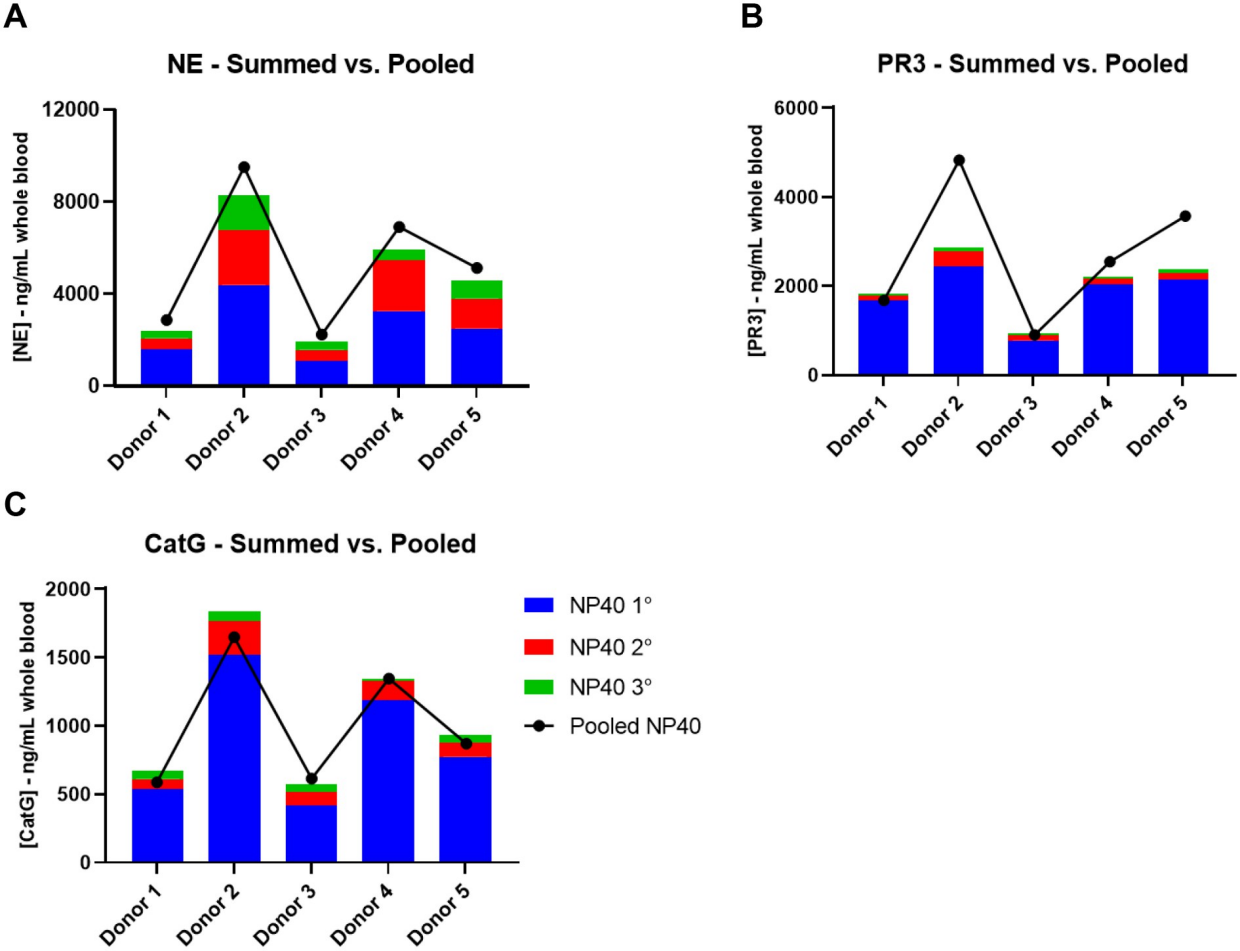
NSP activity recovered from individual WBC lysis cycles versus pooled wbc lysates for NP40. The bars represent the individual lysate measurements which were summed, while the line represents the pooled lysate measurement accounting for the removal of the samples on the total recovery.

**Fig 4:**
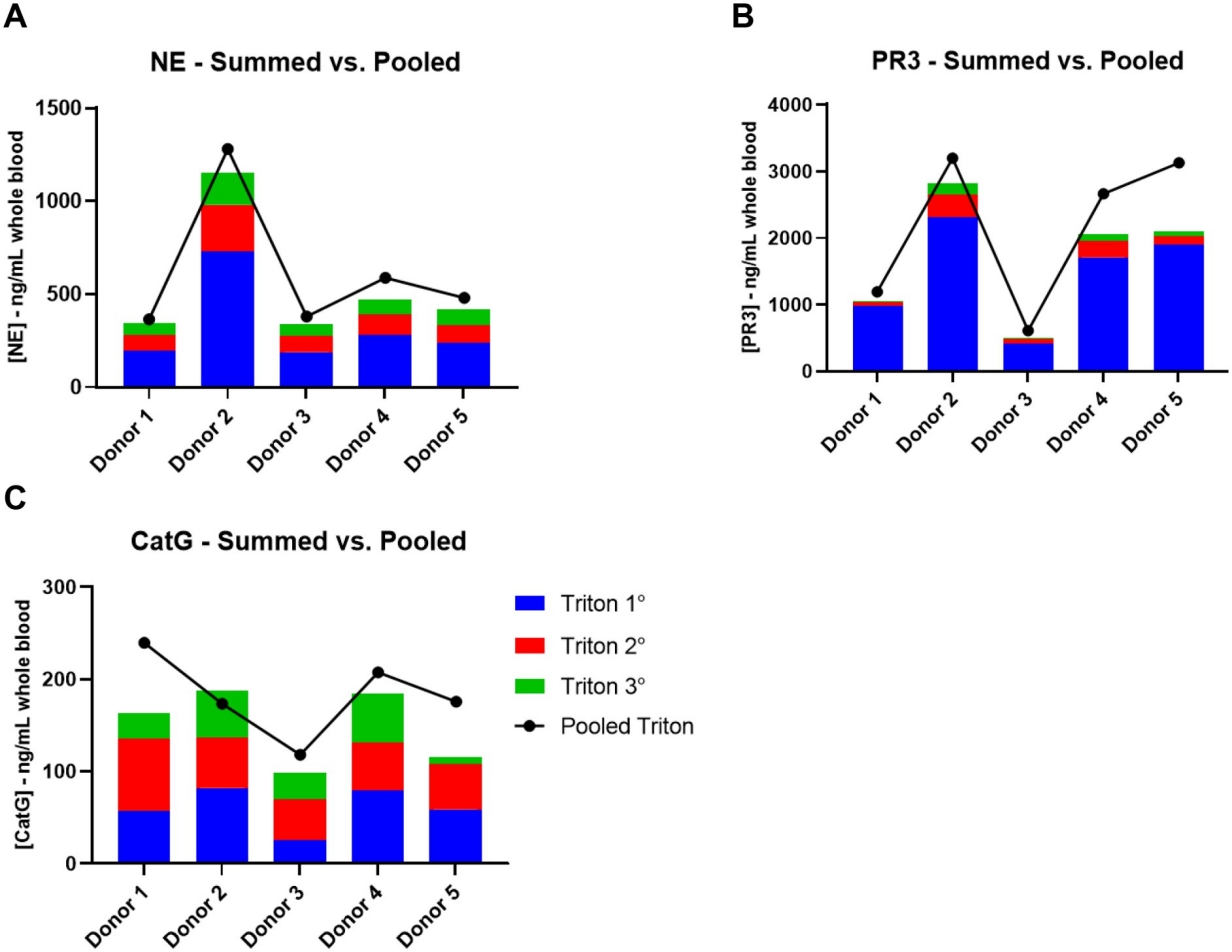
NSP recovery from individual WBC lysis cycles versus pooled WBC lysates for 0.02% Triton X-100. The bars represent the individual lysate measurements which were summed, while the line represents the pooled lysate measurement accounting for the removal of the samples on the total recovery.

In addition to measuring the pooled lysate fractions, individual lysate fractions from the primary, secondary and tertiary lysis cycles were also measured for conditions C and D. The purpose was twofold: (1) to compare the NSP recovery from the sum of the individual lysate fractions to that for the pooled lysate fractions, which could validate the pooled lysis approach; and (2) to determine if additional lysis steps may need to be incorporated to extract additional meaningful NSP activity. Fig 3 shows the results of the summed vs pooled lysate experiment for each of the NSPs using NP40 described in condition C. A similar outcome was found using Triton X-100 as described in condition D and that is reported in Fig 4. The bars represent the individual lysate measurements which were summed, while the line represents the pooled lysate measurement accounting for the removal of the samples on the total recovery.

The summed and pooled measurements are relatively close to each other and follow the same inter-donor trend, suggesting that pooling lysate fractions is valid for quantitation of NE, PR3 and CatG (Fig 3). Statistical analysis of the average NSP donor measurements determined that there was no difference between the two methodologies (P = 0.2791 for NE; P = 0.9695 for PR3; P = 0.0108 for CatG). Thus, pooling of samples for analysis will reduce assay burden.

Each lysis cycle results in additional recovery of NE with each subsequent extraction, albeit to a lesser extent each time. Primary lysis extracts approximately 57% of the total NE recovered in the three extractions and more than 80% for PR3 and CatG. Secondary and tertiary lysis results in approximately 28 and 15% total NE extracted, respectively, whereas tertiary lysis extracts less than 5% additional PR3 and CatG. Given this data, triple lysis may be beneficial for near complete extraction of NE and help quantify the pharmacodynamic (PD) biomarker but contribute very little to PR3 or CatG biomarkers since there is near complete extraction after two lysis steps. These data indicate that further lysis cycles are unlikely to meaningfully increase the NSP extraction recovery efficiency.

### Comparison of the cell pellet extraction method to the zymosan stimulation method for NSP recovery

Boiling zymosan did not significantly affect the NSP recovery compared to the un-boiled zymosan counterpart for the majority of NSPs; therefore, since the un-boiled zymosan preparation is a simpler procedure, the NP40 cell pellet lysis results were compared to the un-boiled zymosan results. Overall, NP40 lysis shows greater recovery of active NSP than zymosan stimulation by ~13.3-fold for NE, by ~2.9-fold for PR3 and by ~283-fold for CatG (Table 5). The NP40 lysis method also had greater inter-donor consistency than the zymosan method for PR3 and CatG, which is most likely attributed to the high assay interference at play in the plasma matrix of the zymosan assay. While Triton X-100 lysis was not as effective as NP40, the Triton X-100 process also yielded greater recoveries of active NSPs than the zymosan stimulation method.

**Table 5:** Recovery of active NSP for the cell pellet extraction vs. zymosan stimulation procedures.

	Ratio of Cell Pellet [NSP] to Zymosan [NSP]		
	[NE]*	[PR3]*	[CatG]*
**Condition A (0.05% NP40)**	10.86	3.94	322
**Condition B (0.05% NP40)**	13.86	2.26	240
**Condition C (0.05% NP40)**	15.18	2.50	286
**Mean for Three NP40 Conditions**	13.3	2.9	283
**Condition D (0.02% Triton X-100)**	1.87	1.97	40.7

*Average ratio for 5 donors.

## Discussion

Neutrophil serine proteases play an essential role in intracellular pathogen destruction and are released during neutrophil activation, contributing to inflammatory regulation and modulation. Despite their critical role in host defense, an excessive neutrophil accumulation and activation (and therefore secretion of active NSPs) can cause damaging inflammation and tissue matrix destruction, as observed in chronic inflammatory diseases [1,2]. In one such disease termed non-cystic fibrosis bronchiectasis (NCFBE), this process has been characterized as a vicious cycle of infection and inflammation [21]. The activity of NE is elevated in lung sputum samples of subjects with NCFBE, and higher NE levels have been associated with a more rapid decline in lung function, increased episodes of pulmonary exacerbations (PEs), and greater mortality [20]. A therapeutic intervention targeting inhibition of DPP1 and leading to a reduction in the destructive inflammatory response could thus help to break the vicious cycle of infection and inflammation and reduce the frequency of PEs. Supporting this thesis, a 6-month Phase 2 trial of brensocatib, a reversible DPP1 inhibitor, demonstrated a dose-dependent reduction in NE levels in sputum, which were indeed associated with a statistically significant prolongation in the time to first PE, and a reduction in the frequency of PEs [19]. However, NSP levels from the circulating neutrophils of this trial have not been published.

Two NSP recovery methodologies have been reported to evaluate these biomarkers in clinical samples; i.e., a zymosan stimulation method [5,7] and a cell pellet extraction method [12,16,17]. Zymosan, a cell wall polysaccharide from *Saccharomyces cerevisiae*, has historically been used to stimulate a physiologically representative inflammatory response to infection. However, it is unclear whether it is fully effective at releasing both the NSPs stored within the neutrophil granules and those which are bound on the surface. Additionally, since this method does not isolate the WBC from the other blood components, endogenous inhibitors of serine proteases may be present in the sample and interfere with NSP activity measurements. It is therefore important to control and normalize for any endogenous serine protease inhibitors by matching the reference standard enzyme matrix and dilution with the standard matrix and dilution.

In contrast, surfactants may be better able to break the lipid-lipid and lipid-protein interactions on the surfaces of the neutrophils to release membrane-bound NSPs, and in combination with appropriate mechanical agitation, may also be more effective at releasing granule-associated NSPs. Furthermore, isolating the WBC from the whole blood eliminates potential endogenous inhibition from plasmatic serine protease inhibitors after NSP extraction from the granules. Given the goal to understand the effect of a pharmacological agent that inhibits NSP activation, an extraction process that recovers a more complete NSP profile is more likely to inform on the effectiveness of that intervention compared to one with low NSP recovery. To determine which method optimally extracts these biomarkers for disease and PD analysis, we conducted a head-to-head study assessing the levels of recovered NSPs and the ease of protocol transfer to clinical and contract research organization (CRO) sites in charge of processing and assaying the samples. In our head-to-head studies, surfactant extraction of NSPs using NP40 was clearly superior to that using Triton X-100, or to the zymosan stimulation method. Our studies confirm the importance of understanding the limitations and benefits of any NSP assay when used in an *in vivo* or clinical setting.

For any extraction methodology, it may be impossible to precisely quantify the percentage of NSP activity recovered because the starting activity is unknown. However, the effectiveness of various extraction methodologies can be estimated relative to each other. In these head-to-head studies on human donor samples, the NP40 lysis procedure was much more effective at extracting active NSPs than either the Triton X-100 lysis or the zymosan stimulation methods; however, it is still unknown what percentage of NSP activity was recovered for any of the methods. By conducting repeated cycles of extraction using the same buffer, one can estimate the value of continued extraction cycles, and in these studies, there appears to be diminishing returns after three cycles. But that still does not inform on the total NSP activity available for recovery—there may be pockets of NSP activity that are still unrecovered after multiple extraction cycles using a specific surfactant or mechanical intervention, as is certainly evident comparing the much lower total NSP recovery using Triton X-100 in comparison to NP40. NP40 was able to recover large pockets of NSP activity that were inaccessible to Triton X-100, but there is no way to know if there were other pockets of NSPs that remained elusive even to NP40 recovery.

Both the zymosan and surfactant lysis procedures have been used to recover NE levels from clinical samples. The zymosan stimulation method was used in a Phase I clinical trial evaluating brensocatib (formerly known as AZD7986) and it showed a dose-dependent reduction of NE [5]. This drug candidate would also be expected to reduce active PR3 and CatG levels as DPP1 is an upstream activator of NE, PR3 and CatG, but these biomarkers were not reported [5]. In our head-to-head study, the zymosan stimulation methodology showed good repeatability for NE with < 5% variation for duplicate samples, but poor repeatability for PR3 and CatG. Moreover, all three NSPs were recovered at substantially lower levels compared to the NP40 cell pellet extraction method. As only a small portion of NSPs is released during the zymosan-induced degranulation process, it is reasonable to question whether the zymosan assay can be used to accurately compare NE levels among individuals or expect to observe therapeutic-induced changes in NE levels. However, in our studies, the rank order of NE levels in individuals was similar using both the zymosan methodologies and the NP40 lysis conditions with donor 2 having the highest NE and donor 3 having the lowest NE. This suggests that the zymosan-stimulation methodology may have some value in assessing differences in NE levels, which takes the neutrophil degranulation process into consideration.

In a Phase 1 clinical trial evaluating an irreversible DPP1 inhibitor (GSK2793660) dosed over 21 days, a 20 to 30% reduction in levels of NE, CatG and PR3 was reported within 1 hour of the first dose of GSK2793660 compared to placebo, which was unexpected given that the effect of DPP1 inhibition occurs in the maturing neutrophils in the bone marrow and changes in NSP levels in whole blood should not be observed for 2–3 weeks [1,2]. Also surprising to the authors and without good explanation was that no further reduction in NSP levels was observed over the following 21-day period. The NSP levels from subjects in that trial were obtained by cell pellet lysis using 0.2% Triton X-100. Given the incomplete recoveries reported in our studies using 0.02% Triton X-100, it is possible that any true NSP signal in response to treatment was masked by the relatively poor extraction efficiency of the Triton X-100 lysis method. Furthermore, the trial size was very small with only five subjects in the placebo group and ten in the active group. Given the observation reported herein that the donor with the highest NSP levels had four-fold greater NSP levels than the donor with the lowest NSP levels, it is possible that the observed 20–30% decline in NSP levels in the active group compared to the placebo group simply reflected differences in baseline levels among the subjects. Thus, comparison of NSP levels between a small number of active and treatment subjects may represent mere donor-to-donor variability.

To circumvent this shortcoming, we evaluated the effectiveness of multiple extraction conditions on “identical” samples, i.e., from the same donor. Thus, it was important to have an adequate volume of blood from each donor to allow for the study of each extraction condition. One limitation of this study was the 24 mL volume of blood that was approved for collection from each donor. That volume allowed for two replicates to be performed on condition A of the NP40 procedure, as well as both the un-boiled and boiled zymosan conditions and the saline control analysis. The condition A replicates confirmed that the generation of the WBC pellets was a repeatable process, with the resulting NSP values demonstrating tight CVs. The zymosan stimulation assay also demonstrated acceptable repeatability for NE, but not for PR3 or CatG, suggesting that the variability for the PR3 and CatG replicates was not due to irregularities during sample generation, but likely due to variability inherent to those assays. While conditions B and C were only done in single replicates, they were essentially repeats of each other and the data was comparable between the two conditions. Thus, only condition D using Triton X-100 did not have a replicate. This limitation was acceptable because it was known prior to protocol conduct that the extraction efficiency of Triton X-100 was inferior to NP40 and so the repeatability of that condition was of lesser importance.

The purpose of this study was to compare various methodologies to recover active NSPs from whole blood samples. For each donor, the neutrophil counts in each replicate aliquot should be comparable allowing for comparison of the NSP extraction methodologies to each other on a donor-by-donor basis. However, one limitation is that while up to a four-fold range in recovered NE activity was observed across the five donors, it is not known whether those differences were due entirely to changes in relative NE activity, or whether changes in neutrophil counts may also have contributed to the extent of the differences. In considering application of this method to a clinical scenario where the goal would be to monitor NSP activities in an individual over time, or across treatment groups, blood cell counts should be measured to allow for normalization of NSP activity.

Although the zymosan and cell pellet extraction methods both utilize short processing procedures at the clinical site, the cell pellet method has a lengthy processing procedure to lyse the WBC pellet at the CRO lab. Additionally, improper decanting or handling at the clinical site may lead to partial or complete loss of a cell pellet, so training of clinical site personnel may be important for this step. Despite these potential drawbacks, the NP40 cell pellet extraction method showed much greater recovery for all three NSPs compared to the zymosan stimulation method and is therefore recommended. Thus, for evaluation of the effect of pharmacological agents like DPP1 inhibitors on NSP activity, an extraction method that more completely recovers the full complement of NSPs will better inform on the pharmacodynamic response.

## Conclusions

Zymosan stimulation showed consistent experimental replicates for the blood samples from five human donors as well as consistent inter-donor activity for NE. However, it failed to repeatably recover PR3 and CatG for activity assessments. Three cycles of cell pellet extraction using a buffer containing 0.02% Triton X-100 buffer resulted in improved NSP recoveries over zymosan-stimulation by 1.8 times for NE, 2.0 times for PR3 and 41 times for CatG. The most efficient cell pellet extraction process utilized three cycles with a buffer containing 0.05% NP40. This process repeatably extracted all three NSPs with approximately 13.3-fold greater NE, 2.9-fold greater PR3 and 283-fold greater CatG activities than the zymosan stimulation process.

## Supporting information

S1 Fig Evaluation and selection of Triton X-100 concentration for extraction studies. Various concentrations of Triton X-100 NSP were compared for NSP extraction efficiency from donor whole blood that had been purchased commercially from BioIVT. For CatG, 10% Triton X-100 showed high levels of interference (~78%) that resulted in the inability to measure CatG activity in that matrix. It also showed the lowest extraction of NE activity. Thus, exploration of this lysis buffer concentration was not continued despite its promising PR3 extraction efficiency as this studied aimed to determine an extraction buffer and method that could efficiently recover all three NSPs. Triton X-100 at 0.02% was selected for further investigation given its comparable level of extraction to 0.2% and 1% Triton X-100 for NE and PR3, and superior extraction for CatG.
(DOCX)

S2 Fig Example kinetic profiles depicting the linear regions determined and used for calculation of NSP activities. (DOCX)

S3 Fig Comparison of select NSP assay standards with sample diluent (enzyme buffer) versus plasma: Enzyme buffer standard diluent matrix at 1:1 (NE), 1:9 (PR3), and 1:1 (CatG), which matched the sample dilution. Plasma diluted in standard matrix greatly interfered with the NE and PR3 enzymatic fluorogenic assays, resulting in >85% reduction in the measured RFU/min, but did not substantially interfere with the CatG enzymatic chromogenic assay.
(DOCX)

S1 Table Recovered NSP activity for the zymosan-stimulation method (Mean ± SD, n = 5 donors). (DOCX)

S2 Table Donor replicates of NSP activity after zymosan-stimulation (NSP ng/mL whole blood, mean and %CV for each donor replicate, n = 2). (DOCX)

S3 Table Recovered NSP activity for the cell pellet extraction method (Mean ± SD, n = 5 donors). (DOCX)

S1 Data (XLSX)

## Abbreviations

Abz: 2-Aminobenzoyl or anthraniloyl
AMC: 7-amino 4-methyl coumarin
AZ: AstraZeneca
CatG: Cathepsin G
CRO: Contract research organization
CV: Coefficient of variation
DPP1: Dipeptidyl peptidase I
ELISA: Enzyme-linked immunosorbent assay
fMLP: N-formyl-L-methionyl-L-leucyl-L-phenylalanine
HRP: Horseradish peroxidase
LOD: Limit of detection
MOA: Mechanism of action
NCFBE: Non-cystic fibrosis bronchiectasis
NE: Neutrophil elastase
NET: neutrophil extracellular trap
NP40: Nonidet P-40 Substitute
NSP: Neutrophil serine protease
PBS: Phosphate buffered saline
PD: Pharmacodynamic
PE: Pulmonary exacerbation
PK: Pharmacokinetic
pNA: p-Nitroaniline
PR3: Proteinase 3
RBC: Red blood cell
RFU: Relative fluorescence units
TMB: Tetramethylbenzidine
WBC: White blood cell
