# Supplementary figures and images for: An optimized method of extracting and quantifying active Neutrophil serine proteases from human whole blood cells
Source: PLoS One. 2022 Aug 31;17(8):e0272575. doi: 10.1371/journal.pone.0272575 (PMC9432755; doi:10.1371/journal.pone.0272575)

| A  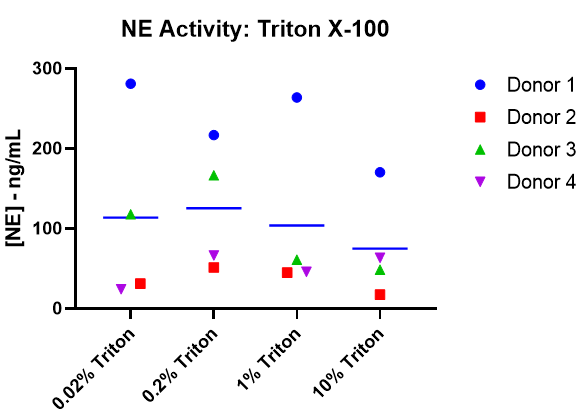 | B  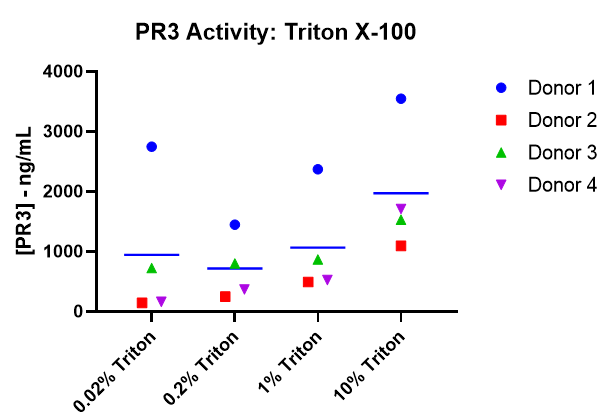 |
| --- | --- |
| C  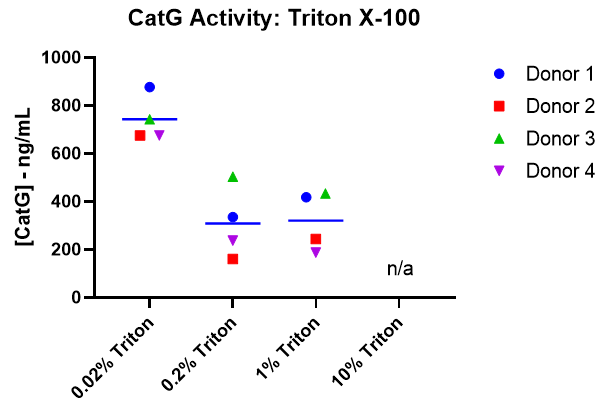 | |

Supplement: S1 Fig — Various concentrations of Triton X-100 NSP were compared for NSP extraction efficiency from donor whole blood that had been purchased commercially from BioIVT. For CatG, 10% Triton X-100 showed high levels of interference (~78%) that resulted in the inability to measure CatG activity in that matrix. It also showed the lowest extraction of NE activity. Thus, exploration of this lysis buffer concentration was not continued despite its promising PR3 extraction efficiency as this studied aimed to determine an extraction buffer and method that could efficiently recover all three NSPs. Triton X-100 at 0.02% was selected for further investigation given its comparable level of extraction to 0.2% and 1% Triton X-100 for NE and PR3, and superior extraction for CatG. (DOCX) [file pone.0272575.s001.docx]

| **A**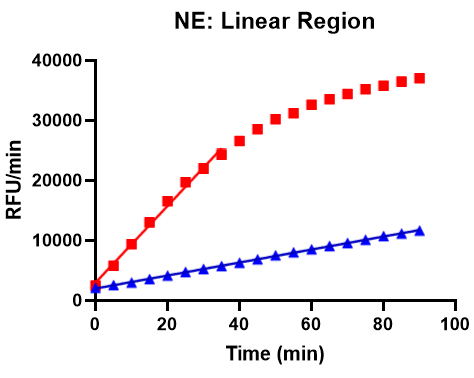 | **B**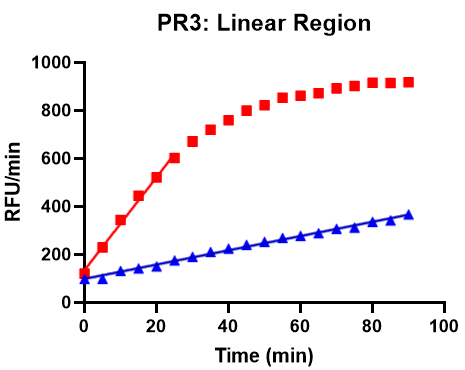 |
| --- | --- |
| **C**  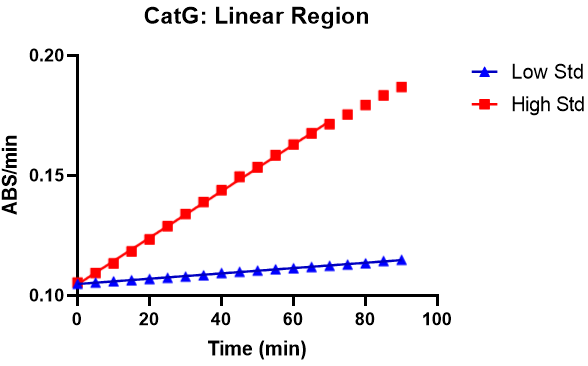 | |

Supplement: S2 Fig — (DOCX) [file pone.0272575.s002.docx]

| **A**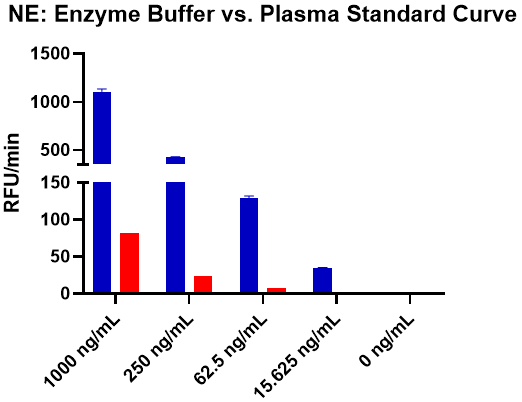 | **B**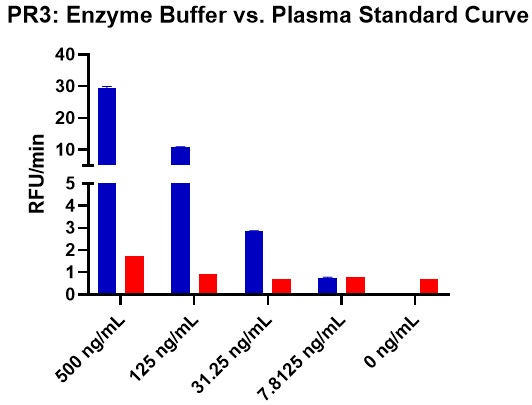 |
| --- | --- |
| **C**  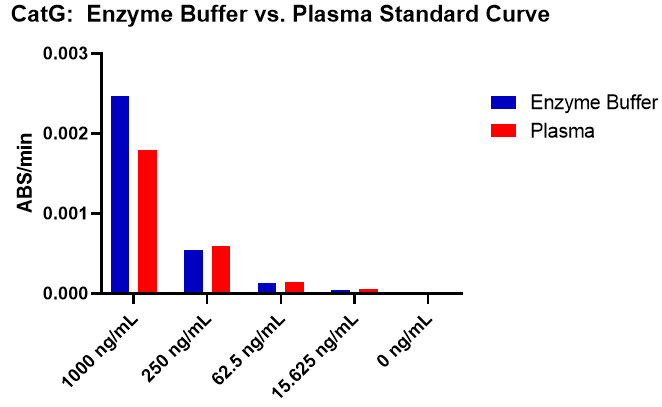 | |

Supplement: S3 Fig — Plasma diluted in standard matrix greatly interfered with the NE and PR3 enzymatic fluorogenic assays, resulting in >85% reduction in the measured RFU/min, but did not substantially interfere with the CatG enzymatic chromogenic assay. (DOCX) [file pone.0272575.s003.docx]
