# Supplementary material for: An optimized method of extracting and quantifying active Neutrophil serine proteases from human whole blood cells
Source: PLoS One. 2022 Aug 31;17(8):e0272575. doi: 10.1371/journal.pone.0272575 (PMC9432755; doi:10.1371/journal.pone.0272575)
Supplement: S1 Table — (DOCX) [file pone.0272575.s004.docx]

S1 Table: Recovered NSP Activity for the Zymosan-Stimulation Method (Mean ± SD, n=5 Donors).

|  | **[NSP] – ng/mL whole blood** | | |
| --- | --- | --- | --- |
|  | Saline | Un-boiled Zymosan | Boiled Zymosan |
| ***NE*** | 6.13 ± 6.27 | 294.99 ± 65.04 | 328.47 ± 90.07 |
| ***PR3*** | 268.53 ± 265.52 | 1086.96 ± 221.17 | 751.00 ± 535.24 |
| ***CatG*** | 19.99 ± 10.68 | 31.54 ± 11.30 | 41.92 ± 14.68 |
