# Supplementary material for: An optimized method of extracting and quantifying active Neutrophil serine proteases from human whole blood cells
Source: PLoS One. 2022 Aug 31;17(8):e0272575. doi: 10.1371/journal.pone.0272575 (PMC9432755; doi:10.1371/journal.pone.0272575)
Supplement: S2 Table — (DOCX) [file pone.0272575.s005.docx]

S2 Table: Donor Replicates of NSP Activity After Zymosan-Stimulation (NSP ng/mL whole blood, mean and %CV for each donor replicate, n=2).

|  |  | ***Donor 1*** | | ***Donor 2*** | | ***Donor 3*** | | ***Donor 4*** | | ***Donor 5*** | |
| --- | --- | --- | --- | --- | --- | --- | --- | --- | --- | --- | --- |
|  |  | Mean | %CV | Mean | %CV | Mean | %CV | Mean | %CV | Mean | %CV |
| ***NE*** | **Saline** | 2.3 | 141.2 | <LOD | n/a | 5.3 | 141.4 | 6.8 | 141.4 | 16.3 | 103.8 |
|  | **Un-boiled Zymosan** | 248.7 | 4.8 | 362.1 | 1.0 | 214.4 | 0.0 | 293.4 | 4.6 | 356.4 | 1.6 |
|  | **Boiled Zymosan** | 287.5 | 3.4 | 450.4 | 1.9 | 217.5 | 0.7 | 303.8 | 2.8 | 383.2 | 2.8 |
| ***PR3*** | **Saline** | 300.1 | 49.6 | <LOD | n/a | 588.5 | 73.0 | 454.0 | 76.4 | <LOD | n/a |
|  | **Un-boiled Zymosan** | 928.7 | 24.3 | 906.86 | 136.7 | 1062.0 | 8.7 | 1457.8 | 21.4 | 1079.5 | 9.1 |
|  | **Boiled Zymosan** | 1083.9 | 5.9 | <LOD | n/a | 1366.3 | 8.9 | 843.5 | 22.1 | 461.3 | 83.2 |
| ***CatG*** | **Saline** | 3.9 | 0.0 | 22.2 | 14.5 | 33.2 | 15.8 | 23.5 | 38.1 | 17.2 | 0.0 |
|  | **Un-boiled Zymosan** | 15.1 | 39.3 | 39.2 | 14.7 | 36.9 | 0.0 | 24.7 | 59.3 | 41.8 | 4.8 |
|  | **Boiled Zymosan** | 16.8 | 20.6 | 50.4 | 13.1 | 40.8 | 23.4 | 50.8 | 19.0 | 50.8 | 5.0 |

*LOD for NE, PR3 and CatG activity assays were 15.625, 7.8125, and 7.8125 ng/mL, respectively
