# Supplementary material for: An optimized method of extracting and quantifying active Neutrophil serine proteases from human whole blood cells
Source: PLoS One. 2022 Aug 31;17(8):e0272575. doi: 10.1371/journal.pone.0272575 (PMC9432755; doi:10.1371/journal.pone.0272575)
Supplement: S3 Table — (DOCX) [file pone.0272575.s006.docx]

S3 Table: Recovered NSP Activity for the Cell Pellet Extraction Method (Mean ± SD, n=5 Donors).

|  | **[NSP] – ng/mL whole blood** | | | |
| --- | --- | --- | --- | --- |
|  | Condition A | Condition B | Condition C | Condition D |
| **NE** | 3319.19 ± 1974.77 | 4211.31 ± 2403.67 | 4620.62 ± 2615.62 | 546.21 ± 344.65 |
| **PR3** | 3391.58 ± 2075.11 | 1961.85 ± 1119.31 | 2047.99 ± 717.75 | 1704.56 ± 922.22 |
| **CatG** | 1139.90 ± 571.16 | 888.83 ± 462.48 | 1072.47 ± 520.56 | 150.14 ± 40.51 |
